# Supplementary material for: Wearable Noninvasive Glucose Sensor Based on CuxO NFs/Cu NPs Nanocomposites
Source: Sensors (Basel). 2023 Jan 7;23(2):695. doi: 10.3390/s23020695 (PMC9865846; doi:10.3390/s23020695)
Supplement: Supplementary file 1 [file sensors-23-00695-s001.zip › sensors-2130952-supplementary.pdf]

Wearable noninvasive glucose sensor based on Cu_xO NFs/Cu NPs nanocomposites

Zhipeng Yu[1], Huan Wu[1], Zhongshuang Xu[1], Zhimao Yang[1], Jian Lv[1], Chuncai Kong[1]*

1.Ministry of Education Key Laboratory for Non-equilibrium Synthesis and Modulation of Condensed Matter, Shaanxi Province Key Laboratory of Advanced Functional Materials and Mesoscopic Physics, School of Physics, Xi'an Jiaotong University, Xi'an, 710049, Shaanxi, China;

*Corresponding Author: kongcc@xjtu.edu.cn (Chuncai Kong)

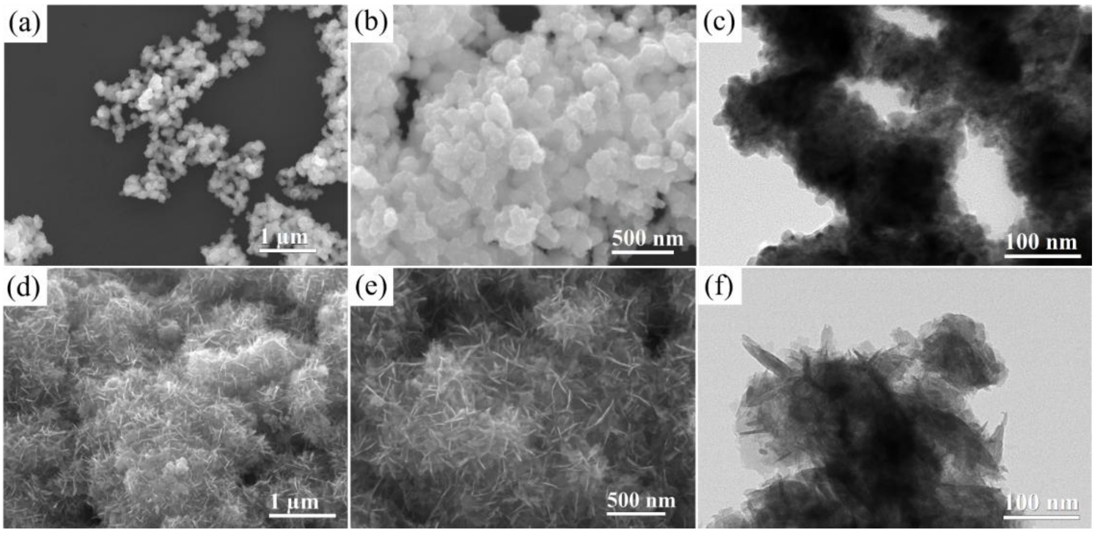

Figure S1. SEM and TEM images of **(a-b)**, **(c)** Cu NPs and **(d-e)**, **(f)** CuO NFs/Cu NPs nanocomposites.

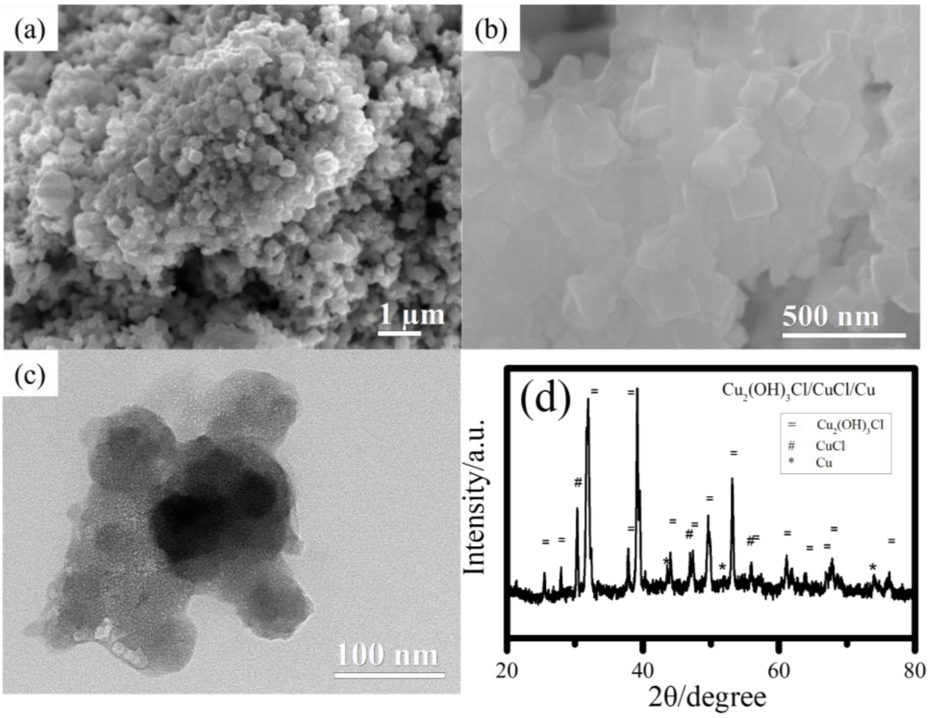

Figure S2. (a, b) SEM, **(c)** TEM images and **(d)** XRD pattern of intermediate product $Cu_2(OH)_3Cl$/CuCl/Cu NPs nanocomposite.

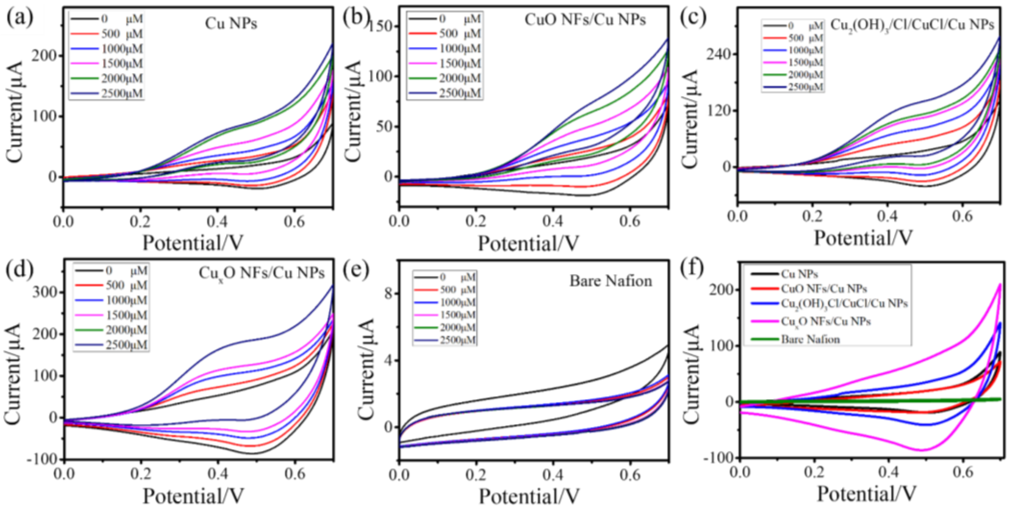

Figure S3. CVs performed in varied glucose concentration with **(a)** Cu NPs, **(b)** CuO NFs/Cu NPs nanocomposites, **(c)** $Cu_2(OH)_3Cl$/CuCl/Cu NPs nanocomposite, **(d)** Cu_xO NFs/Cu NPs nanocomposites, **(e)** bare Nafion at the 100 mV/s scanning rate. **(f)** CVs performed with different samples without the presence of glucose.

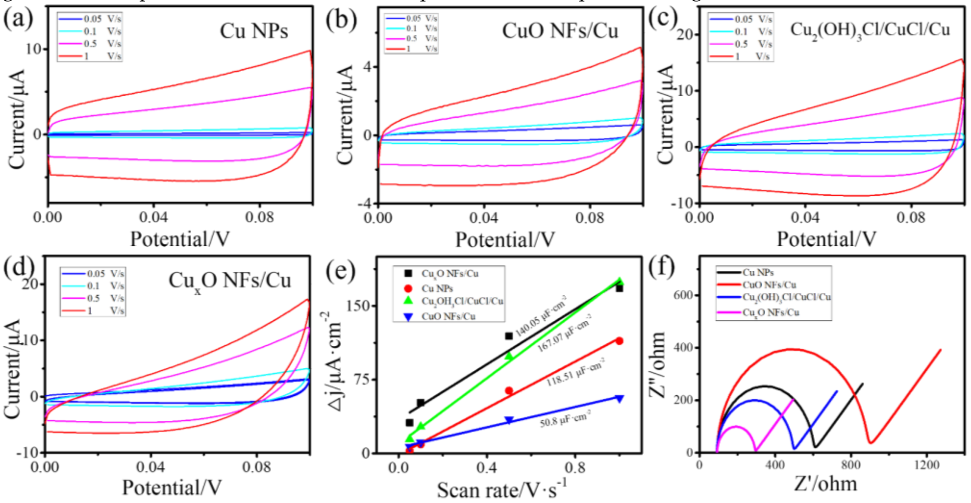

Figure S4. CVs performed in varied scan rates for ECSA measurement with **(a)** Cu NPs, **(b)** CuO NFs/Cu NPs nanocomposites, **(c)** $Cu_2(OH)_3Cl$/CuCl/Cu NPs nanocomposites, **(d)**Cu_xO NFs/Cu NPs nanocomposites. **(e)** The extracted calibration curves of a current response versus the scan rate with different samples. **(f)** EIS fitting performed with different samples without glucose.

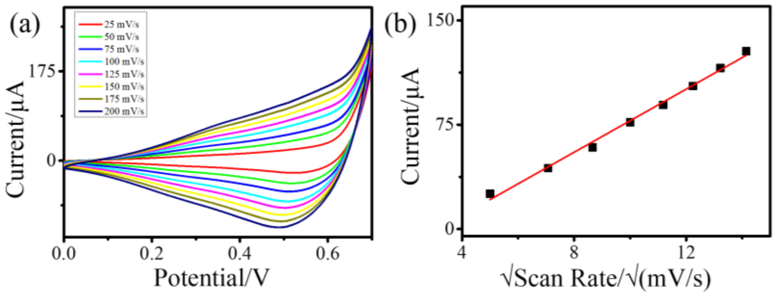

Figure S5. **(a)** CV performed in 1.0 mM glucose with variation of the scanning rate on Cu_xO NFs/Cu NPs nanocomposites and **(b)** Oxidation current density at +0.55 V on the speed of scanning rate.

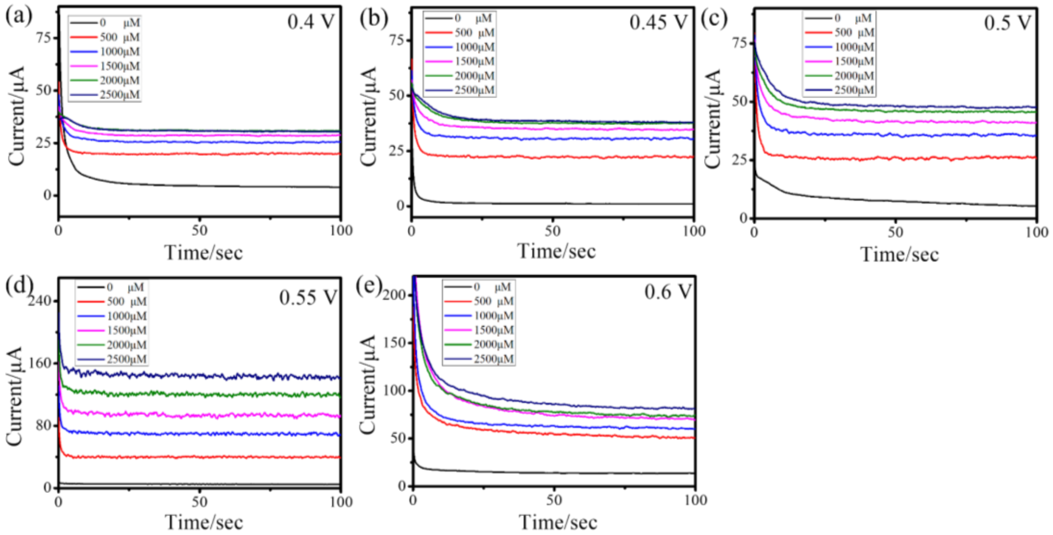

Figure S6. Amperometric response of Cu_xO NFs/Cu NPs nanocomposite-based sensor by a stepwise increase of the glucose concentration with **(a)** +0.4 V, **(b)** +0.45 V, **(c)** +0.5 V, **(d)** +0.55 V, **(e)** +0.6 V.

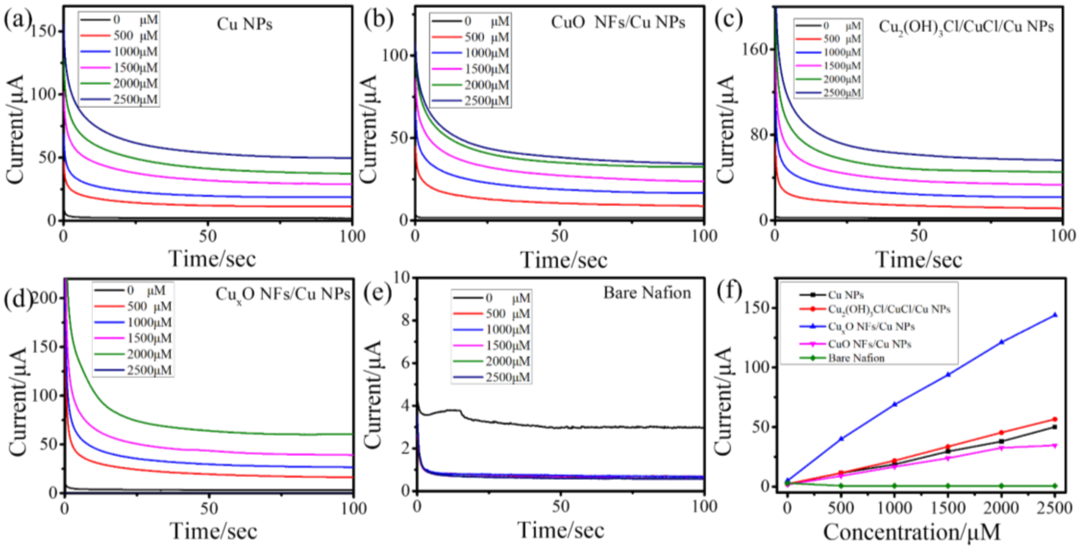

Figure S7. Amperometric response by a stepwise increase of the glucose concentration with **(a)** Cu NPs, **(b)** CuO NFs/Cu NPs nanocomposite, **(c)** $Cu_2(OH)_3Cl$/CuCl/Cu NPs nanocomposite, **(d)** Cu_xO NFs/Cu NPs nanocomposite, **(e)** bare Nafion and **(f)** the extract amperometric curve of three sensors.
